# Impact of Spread Through Air Spaces (STAS) on Recurrence and Surgical Trends in Stage I Non‐Small Cell Lung Cancer: A Real‐World Cohort Study

**DOI:** 10.1002/kjm2.70061

**Published:** 2025-06-17

**Authors:** Cheng‐Hao Chuang, Yu‐Wei Liu, Wei‐An Lai, Yi‐Wen Shen, Shih‐Yu Kao, Yu‐Ching Wei, Chu‐Chun Chien, Hui‐Yang Hung, Jui‐Ying Lee, Min‐Fang Chao, Jen‐Yu Hung, Inn‐Wen Chong, Chih‐Jen Yang

**Affiliations:** ^1^ Division of Pulmonary and Critical Care Medicine, Department of Internal Medicine Kaohsiung Medical University Hospital, Kaohsiung Medical University Kaohsiung Taiwan; ^2^ Graduate Institute of Medicine, College of Medicine, Kaohsiung Medical University Kaohsiung Taiwan; ^3^ Division of Thoracic Surgery, Department of Surgery Kaohsiung Medical University Hospital, Kaohsiung Medical University Kaohsiung Taiwan; ^4^ Department of Pathology Kaohsiung Medical University Hospital, Kaohsiung Medical University Kaohsiung Taiwan; ^5^ Cancer Center, Kaohsiung Medical University Hospital, Kaohsiung Medical University Kaohsiung Taiwan; ^6^ Department of Pathology Kaohsiung Medical University Gangshan Hospital Kaohsiung Taiwan; ^7^ Department of Radiology Kaohsiung Medical University Hospital, Kaohsiung Medical University Kaohsiung Taiwan; ^8^ School of Post‐Baccalaureate Medicine College of Medicine, Kaohsiung Medical University Kaohsiung Taiwan

**Keywords:** LDCT, recurrent free survival, spread through air spaces, Stage I NSCLC, surgery

## Abstract

Non‐small cell lung cancer (NSCLC) is the most common form of lung cancer, which remains a leading cause of cancer mortality worldwide. Early detection, particularly at Stage I, is critical for improving survival outcomes. Low‐dose computed tomography (LDCT) has increased the detection of small asymptomatic tumors, resulting in an earlier diagnosis and facilitating less invasive surgical approaches such as segmentectomy and wedge resection, which preserve lung function while offering survival outcomes comparable to lobectomy. However, the risk factors for recurrence in Stage I NSCLC remain poorly understood. This retrospective study analyzed 1077 Stage I NSCLC patients treated at Kaohsiung Medical University Hospital from 2010 to 2022. Data including AJCC 7th and 8th editions staging, surgical interventions, and pathological features were analyzed. The proportion of Stage I cases increased significantly from 9.3% in 2010 to 33.8% in 2017, with Stage IA cases increasing from 12.1% in 2018 to 38.2% in 2022. Concurrently, the lobectomy rate decreased from 75% in 2010 to 43.6% in 2022, with more sublobar resections performed. Kaplan–Meier analysis found no significant differences in recurrence‐free survival between lobectomy and sublobar resection with regional lymph node dissection. Cox regression identified spread through air spaces (STAS) as an independent risk factor for recurrence (hazard ratio 2.87, *p* = 0.006), along with male sex, larger tumor size, and visceral pleural invasion. These findings highlight the role of LDCT in early detection and the importance of tailored treatment strategies, particularly addressing STAS, to optimize recurrence‐free survival and improve outcomes of patients with Stage I NSCLC.

## 1 Introduction

Lung cancer continues to be a leading cause of cancer‐related deaths worldwide, with non‐small cell lung cancer (NSCLC) representing the most prevalent type [1]. Early detection and timely interventions are critical for improving survival outcomes in patients with NSCLC, especially for those with Stage I disease. Detecting lung cancer early offers the best chance for curative treatment through surgery [2]. Over the last decade, advances in imaging technologies and surgical techniques have transformed the diagnosis and treatment of early‐stage lung cancer. In particular, the introduction of low‐dose computed tomography (LDCT) screening has significantly impacted clinical practice by enhancing the ability to detect smaller, asymptomatic tumors at an earlier stage [3, 4, 5].

Current evidence strongly supports the role of low‐dose computed tomography (LDCT) in detecting lung cancer at earlier stages than traditional methods. The National Lung Screening Trial (NLST) and other studies showed that LDCT screening in high‐risk populations can reduce lung cancer mortality by approximately 20%, mainly due to earlier detection of Stage I disease [6]. Gierada and Pinsky reported that among patients with Stage I lung cancer, those detected through LDCT screening had better long‐term lung cancer‐specific survival compared to those diagnosed via chest radiography. The improved survival was associated with smaller tumor size and receipt of surgical resection [7]. Real‐world data also confirm that widespread LDCT implementation leads to a stage shift, with more patients diagnosed at Stage I [2, 8, 9, 10, 11].

LDCT screening has expanded across Asia, with national programs in Japan, South Korea, China, and Taiwan. This has contributed to a higher proportion of early‐stage diagnoses. Additionally, biomarkers such as volume doubling time, tumor mutational burden, and circulating tumor DNA may help predict tumor behavior in early‐stage adenocarcinomas detected by LDCT, which show variable growth patterns based on these markers [12, 13].

There has also been a notable evolution in surgical practices for NSCLC. Historically, lobectomy was considered the standard of care for Stage I NSCLC [14]. However, with the increased detection of smaller tumors, there is growing evidence supporting the implementation of minimal surgical interventions, such as segmentectomy and wedge resection, particularly for tumors ≤ 2 cm [15]. These sublobar resections, when combined with regional lymph node dissection (RLND), have been shown to achieve comparable survival outcomes to lobectomy, while preserving more lung tissue and reducing postoperative morbidity. The JCOG0802/WJOG4607L trial [16] and CALGB/Alliance 140503 trial [17] both demonstrated that segmentectomy provided similar overall survival (OS) to lobectomy for small tumors, with the added benefit of better postoperative lung function. These findings have prompted a paradigm shift in surgical management, with an increasing proportion of patients undergoing lung‐sparing procedures, and particularly in those with Stage IA1 and IA2 disease.

Along with the higher proportion of patients being diagnosed at an earlier stage through LDCT screening, there has been a corresponding improvement in overall lung cancer survival outcomes. In our hospital, the number of LDCT screenings increased from just 29 in 2013 to 1900 in 2022, leading to a substantial increase in the proportion of patients diagnosed with Stage I NSCLC. In addition, there has also been a significant increase in the percentage of NSCLC patients receiving surgery as the first‐line treatment over the past decade, from 23.0% in 2011 to 52% in 2020 [11]. Furthermore, surgical practices have evolved, with a greater emphasis on minimal interventions for smaller tumors. However, the impact of this stage shift and associated sublobar resection approach on patient outcomes is still uncertain.

The aim of this study was therefore to assess the real‐world impact of these trends on patients with Stage I NSCLC over the past decade, focusing on staging (IA1, IA2, IA3, and IB), treatment modalities, and recurrence‐free survival (RFS). Understanding the evolving landscape of NSCLC diagnosis and treatment is essential for optimizing patient care and ensuring that clinical practices align with the latest evidence supporting minimal surgical interventions and the benefits of early detection through LDCT.

## 2 Methods

### 2.1 Study Design and Data Collection

This retrospective cohort study utilized data from Kaohsiung Medical University Hospital (KMUH), an 1800‐bed tertiary teaching hospital in Taiwan. Data from the Taiwan National Cancer Registry through 2022 were also obtained to ensure accurate staging and survival outcomes. Data were collected from the hospital records of NSCLC patients from 2010 to 2022.

According to the AJCC 7th Edition Tumor Size Classification (2010 to 2017), Stage I NSCLC was classified into Stage IA and Stage IB, primarily based on a tumor size threshold of 3 cm as follows:

- Stage IA: Tumor size ≤ 3 cm in greatest dimension.
- Stage IB: Tumor size > 3 cm but ≤ 5 cm, or tumors with additional features such as visceral pleural invasion or obstructive pneumonitis.

This classification did not further subdivide Stage IA based on a smaller tumor size. As a result, tumors as small as 1 cm and those approaching 3 cm were grouped together under Stage IA, despite their differing prognoses.

The AJCC 8th Edition Tumor Size Classification (2018–2022) then further subdivides the classification of Stage I, with a focus on tumor size:

- Stage IA1: Tumor size ≤ 1 cm in greatest dimension.
- Stage IA2: Tumor size > 1 cm but ≤ 2 cm in greatest dimension.
- Stage IA3: Tumor size > 2 cm but ≤ 3 cm in greatest dimension.
- Stage IB: Tumor size > 3 cm but ≤ 4 cm, or any tumor size with invasion into the visceral pleura or atelectasis/obstructive pneumonitis.

This study focused on these Stage I NSCLC subgroups: Stage IA and IB from 2010 to 2017, and IA1, IA2, IA3, and IB from 2018 to 2022. To ensure consistency, tumors diagnosed between 2010 and 2017 were retrospectively stratified according to the AJCC 8th edition criteria as: IA1 (≤ 1 cm), IA2 (1–2 cm), IA3 (2–3 cm), and IB (3–4 cm). Surgical interventions included lobectomy with RLND and two sublobar resections: segmentectomy with RLND and wedge resection with RLND. The surgical approach was primarily determined preoperatively based on imaging and patient comorbidities. However, intraoperative frozen section findings occasionally influenced the final decision, particularly for cases with suspected lymph node involvement.

Survival data for the subgroups were analyzed for patients with follow‐ups extending to December 31, 2022. Patients from 2016 to 2018 were selected for the RFS analysis because STAS data were only recorded in the cancer registry beginning in 2016. These cases also had sufficient follow‐up time through December 2022 to allow for meaningful survival analysis. For the Cox regression model, we enrolled all surgically resected Stage I NSCLC patients treated between January 2016 and December 2018, with follow‐up data available through December 2022. Patients were included if they had histologically confirmed Stage I NSCLC, underwent surgical resection, and had complete follow‐up data. The exclusion criteria were missing pathology data, neoadjuvant therapy, non‐curative surgery, or loss to follow‐up before recurrence was diagnosed.

In addition to basic patient characteristics, we categorized tumor size into two groups: ≤ 20 mm and > 20 mm to ≤ 40 mm. Pathological characteristics included lymphovascular invasion and visceral pleural invasion (VPI), which was further defined as invasion beyond the elastic layer (PL1), invasion into the visceral pleural surface (PL2), and invasion into any component of the parietal pleura (PL3). Spread through air spaces (STAS) was also analyzed, defined as detached tumor cells present beyond the main tumor edge in the alveolar spaces, as observed in histopathological examination. This classification was applied retrospectively based on standardized pathological criteria. Surgical approaches were categorized as lobectomy or sublobar resections (segmentectomy and wedge resection).

### 2.2 Statistical Analysis

Descriptive statistics were used to characterize the cohort, including frequencies and percentages for Stage I subgroups and surgical interventions. Kaplan–Meier survival analysis was performed to estimate the probability of RFS, as it effectively handles censored data, making it appropriate for time‐to‐event analyses commonly used in clinical studies.

To evaluate significant differences in tumor size and stage distribution across Stage I subgroups, a one‐way ANOVA was employed. This approach was chosen due to the ability of ANOVA to compare multiple independent groups efficiently. The Kruskal–Wallis test was used to validate and confirm the robustness of the ANOVA findings.

Multivariate Cox proportional hazards regression models were used to identify independent risk factors influencing RFS, while systematically controlling for potential confounding variables including age, sex, tumor size, surgical approach, and pathological characteristics (specifically STAS and pleural invasion). Confounding variables were adjusted in these models to ensure the reliability and validity of our conclusions.

## 3 Results

### 3.1 Changes in Stage I Subgroup Distribution (2011–2017): AJCC 7th Edition

**FIGURE 1:**
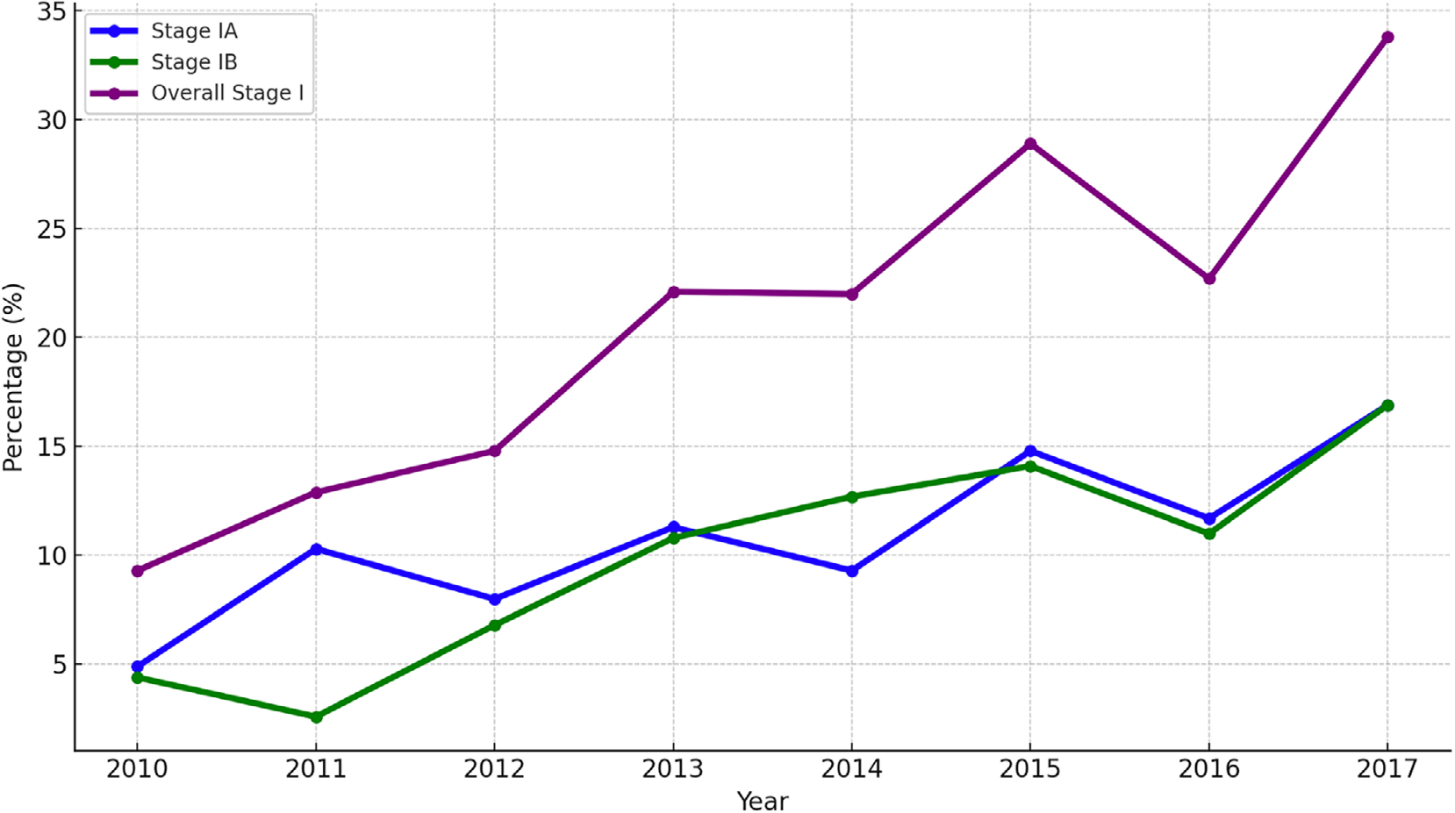
Trend of NSCLC Stage IA, 1B, and overall Stage I diagnoses from 2010 to 2017.

LDCT was introduced in our hospital in 2013, with usage increasing from 29 scans in 2013 to 1900 in 2022. This increase corresponded with a rise in the proportion of Stage I NSCLC diagnoses, particularly Stage IA1. The detection rate of lung cancer by LDCT is defined as the number of newly diagnosed lung cancer patients divided by the total number of LDCT scans performed at our institute. In 2013, the detection rate was 1.2% and 1.8% in 2022 in our previous report [10]. From 2010 to 2017, a total of 437 patients were enrolled. During this period, there was a significant upward trend in the overall percentage of Stage I NSCLC cases (including both Stage IA and Stage IB) (Figure 1). The percentage of Stage IA cases increased from 4.9% in 2010 to 16.9% in 2017, highlighting the improved early detection or diagnosis of smaller tumors (≤ 3 cm). Similarly, the percentage of Stage IB cases increased from 4.4% in 2010 to 16.9% in 2017, reflecting a similar trend for tumors between 3 and 4 cm in size. Overall, Stage I NSCLC cases as a proportion of total diagnoses increased from 9.3% in 2010 to 33.8% in 2017, suggesting a shift toward earlier detection and treatment over time. These trends emphasize the importance of early‐stage diagnosis and the potential benefits of screening programs such as LDCT in identifying NSCLC at earlier, more treatable stages.

### 3.2 Changes in Stage I Subgroup Distribution (2018–2022): AJCC 8th Edition

**FIGURE 2:**
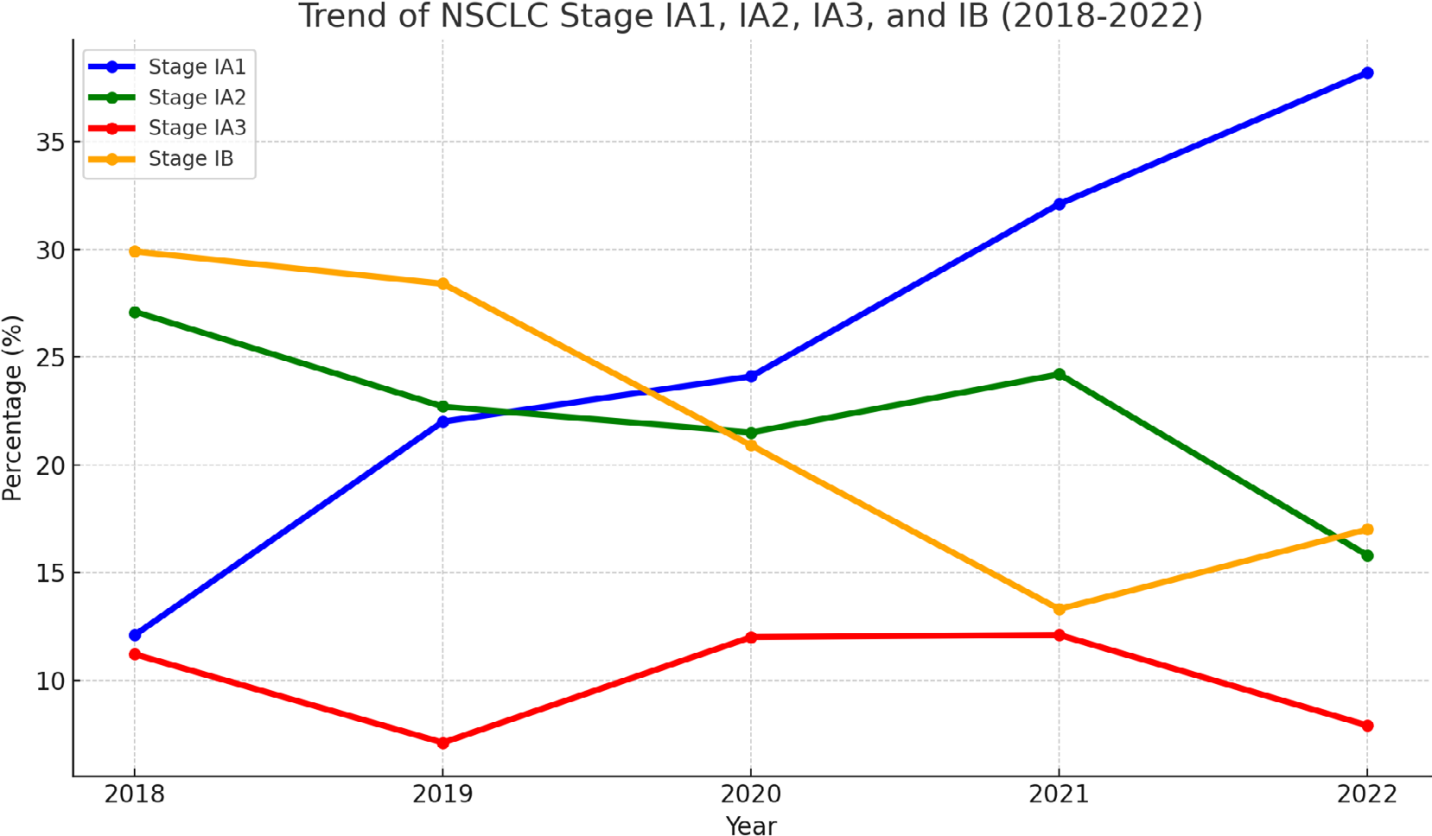
Trend of NSCLC Stage IA1, IA2, IA3, and Stage IB diagnoses from 2018 to 2022.

Between 2018 and 2022, a total of 640 patients were enrolled. A noticeable shift was observed in the distribution of Stage I NSCLC subgroups (Figure 2). The increase in Stage IA1 cases from 12.1% (2018) to 38.2% (2022) aligns with the increase in the number of people screened with LDCT (from 29 in 2013 to 1900 in 2022). The percentage of Stage IA1 cases increased from 12.1% in 2018 to 38.2% in 2022, while the percentage of Stage IA2 cases decreased to 15.8% in 2022. The percentage of Stage IA3 cases slightly decreased from 12.1% to 7.9%, and the percentage of Stage IB cases decreased from 29.9% in 2018 to 17.0% in 2022. The one‐way ANOVA showed statistically significant differences between the Stage I subgroups (*F* value: 8.08, *p* value: 0.00048), indicating a trend toward the earlier detection of smaller tumors, particularly Stage IA1, largely due to the expanded use of LDCT screening.

### 3.3 Trend of Surgery as the First‐Line Treatment for NSCLC Stages I to IV (2011–2020)

**FIGURE 3:**
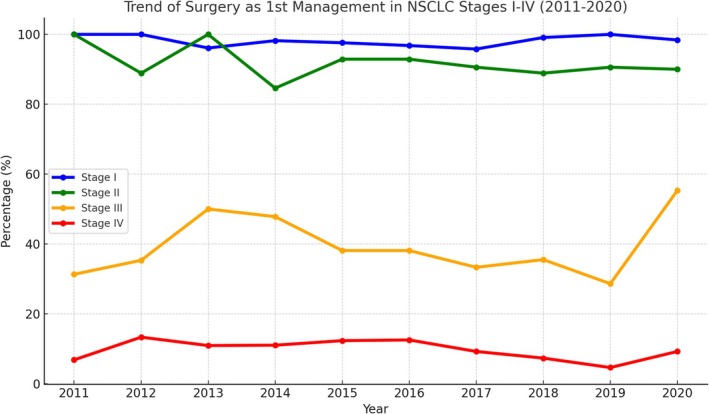
Trend of surgery as the first‐line treatment for NSCLC Stage I–V from 2010 to 2020.

We then analyzed the percentage of patients undergoing surgery as the first‐line treatment across all stages of NSCLC (Figure 3). Surgery was consistently the most common primary treatment for Stage I, followed by Stage II. The percentages of surgical interventions significantly decreased for Stages III and IV, where non‐surgical treatments such as chemotherapy and radiation were more common. The decrease in surgical interventions for advanced NSCLC was most likely due to the increased utilization of targeted therapy and immunotherapy, which offer better survival benefits in advanced cases.

### 3.4 Surgical Interventions for Stage I NSCLC (2010–2022)

**FIGURE 4:**
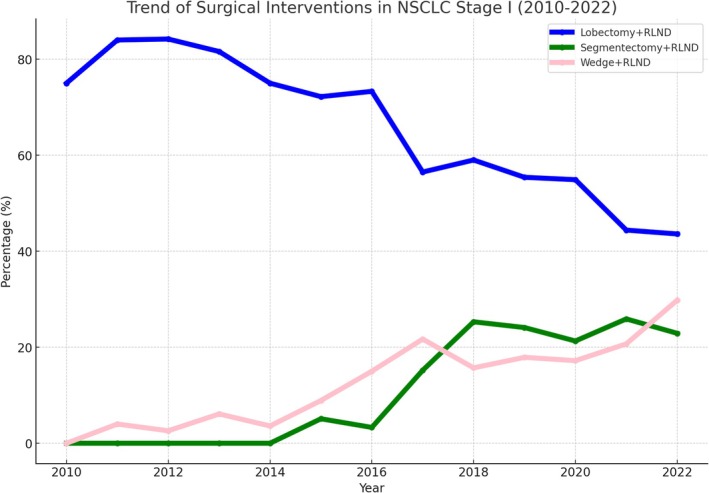
Trend in surgical interventions (lobectomy, segmentectomy, and wedge resection) for Stage I NSCLC from 2010 to 2022.

The trend in surgical interventions for Stage I NSCLC from 2010 to 2022 reflected a shift toward less invasive and lung preserving procedures (Figure 4):

The percentage of lobectomy + RLND decreased from 75% in 2010 to 43.6% in 2022, while the percentage of segmentectomy + RLND increased from 0% to 22.9%. The percentage of wedge resection + RLND increased significantly from 0% in 2010 to 29.8% in 2022. The one‐way ANOVA confirmed statistically significant differences between the trends of these surgical interventions over time (*F* value: 90.53, *p* value: 9.01 × 10^−15^). We used the non‐parametric Kruskal–Wallis test to further validate these results, which confirmed significant differences in the distributions of surgical approaches across the studied periods.

### 3.5 Trend in Surgical Interventions for NSCLC Stage IA1, IA2, IA3, and IB (2018–2022)

**FIGURE 5:**
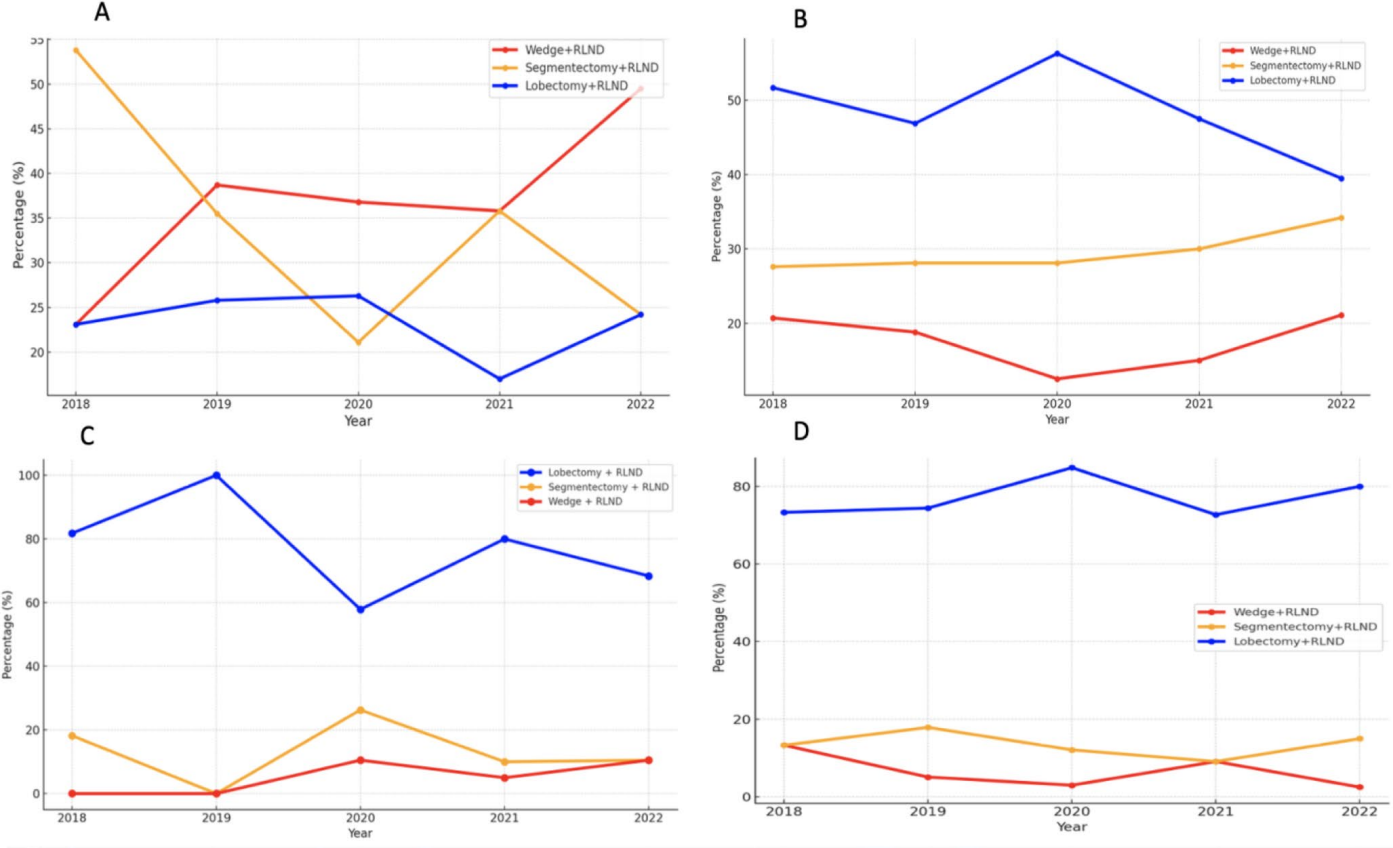
Trends of surgical interventions for NSCLC Stages IA1 (Figure 5A), IA2 (Figure 5B), IA3 (Figure 5C), and IB (Figure 5D) from 2018 to 2022.

A total of 640 Stage I patients were included in the analysis, of whom 610 underwent surgical intervention with RLND. The trend of surgical interventions for NSCLC Stages IA1 (Figure 5A), IA2 (Figure 5B), IA3 (Figure 5C), and IB (Figure 5D), between 2018 and 2022 showed a clear evolution toward more lung‐sparing procedures, with lobectomy still predominant for larger tumors. This shift toward lung‐sparing surgeries, particularly for smaller tumors (IA1 and IA2), highlights the evolving balance between preserving lung function and achieving oncological outcomes. For Stage IB, where the tumor size exceeds 3 cm, lobectomy remained more common due to the higher risks associated with larger tumors.

### 3.6 Analysis of the Type of Surgical Intervention for Stage I NSCLC

**FIGURE 6:**
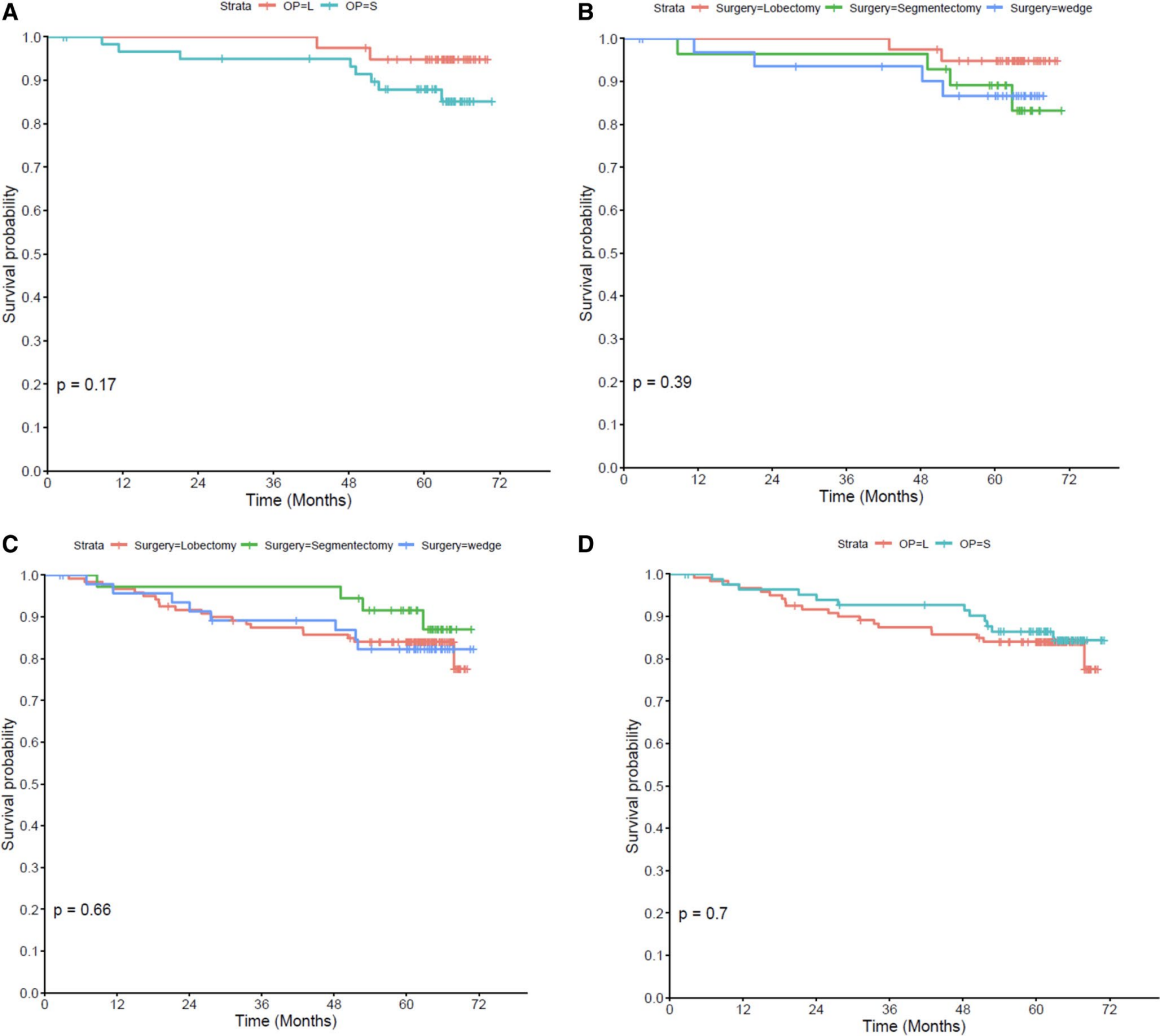
Analysis of the type of surgical intervention for Stage I NSCLC. (A) No significant difference in RFS between the patients with Stage I and a tumor size ≤ 2 cm who received lobectomy and those who received sublobar resection. (B) No significant difference in RFS between the patients with Stage I and a tumor size ≤ 2 cm who received lobectomy, segmentectomy, and wedge resection. (C) No significant difference in RFS between all patients with Stage I who received lobectomy and those who received sublobar resection. (D) No significant difference in RFS between all patients with Stage I who received lobectomy, segmentectomy, and wedge resection.

A total of 207 Stage I NSCLC patients diagnosed between January 2016 and December 2018 who underwent surgical intervention with RLND were included in the RFS analysis. The decision between lobectomy and sublobar resection was influenced by multiple factors. For tumors ≤ 2 cm with predominant ground‐glass opacity (GGO) components, sublobar resection was preferred due to its oncologic equivalence to lobectomy and its advantage in preserving pulmonary function. Conversely, solid‐predominant tumors, particularly those > 2 cm, were more likely to undergo lobectomy to minimize the risk of recurrence. In addition, patient‐related factors including advanced age, compromised pulmonary reserve (e.g., low forced expiratory volume in 1 s [FEV1]), and multiple comorbidities favored sublobar resection to reduce surgical morbidity. After a median follow‐up of 64.5 months, there was no significant difference in 5‐year RFS between the patients with Stage I and a tumor size ≤ 2 cm who received lobectomy and those who received sublobar resection (lobectomy 94.8% vs. sublobar resection 87.8%, *p* = 0.17) (Figure 6A). Similarly, there was no significant difference in 5‐year RFS when comparing specific surgical approaches of lobectomy, segmentectomy, and wedge resection (94.8% vs. 89.1% vs. 86.6%, respectively, *p* = 0.39) (Figure 6B).

Furthermore, no significant difference was identified in 5‐year RFS between all Stage I patients who received lobectomy and sublobar resection (84% vs. 86.4%, *p* = 0.7) (Figure 6C). Similarly, there was no significant difference in 5‐year RFS when comparing specific surgical approaches of lobectomy, segmentectomy, and wedge resection (84% vs. 91.6% vs. 82.3%, *p* = 0.66) (Figure 6D).

### 3.7 Cox Regression Model for RFS

After a median follow‐up of 63.7 months, a total of 207 Stage I NSCLC patients diagnosed between January 2016 and December 2018 were included in this retrospective analysis, with 34 documented cases of recurrence. In the Cox regression model for RFS, univariate analysis identified male sex, larger tumor size (HR 2.36, 95% CI 1.18–4.73, *p* = 0.016), STAS (HR 3.40, 95% CI 1.68–6.88, *p* = 0.001), and visceral pleural invasion (PL2) (HR 5.28, 95% CI 1.93–14.46, *p* = 0.001) as significant risk factors. Multivariate analysis showed that STAS (HR 2.87, 95% CI 1.35–6.07, *p* = 0.006) was the only independent risk factor for recurrence (Table 1). Notably, data on STAS were recorded in the cancer registration system beginning in January 2016.

**TABLE 1:** Cox regression model for recurrence‐free survival in the patients with Stage I lung cancer from January 2016 to December 2018.

Variables	Univariate			Multivariable		
	HR	(95% CI)	*p*	HR	(95% CI)	*p*
Gender						
Female	Reference					
Male	1.62	(0.81, 3.24)	0.175			
Age group						
< 65	Reference					
≧ 65	1.79	(0.89, 3.58)	0.101			
Smoking						
N	Reference					
Y	0.958	(0.41, 2.22)	0.92			
Tumor size (分組)						
≦ 20	Reference			Reference		
> 20, ≦ 40	2.36	(1.18, 4.73)	0.016	1.65	(0.79, 3.46)	0.183
Lymph vascular invasion^2^						
N	Reference			Reference		
Y	2.21	(0.85, 5.74)	0.104			
STAS^1^						
N	Reference			Reference		
Y	3.40	(1.68, 6.88)	0.001	2.87	(1.35, 6.07)	0.006
Pleural invasion						
PL 0	Reference					
PL 1	1.37	(0.63, 2.96)	0.424			
PL 2	5.28	(1.93, 14.46)	0.001			
Adenocarcinoma subtype						
Minimally invasive, non‐mucinous	Reference					
Acinar	1.93	(0.26, 14.40)	0.521			
Lepidic	0.39	(0.02, 6.21)	0.503			
Others^3^	2.05	(0.26, 16.01)	0.495			
Operation type						
Lobar	Reference					
Sublobar^4^	0.87	(0.42, 1.78)	0.702			

*Note*: ^1^STAS: spread through air space. ^2^Visceral pleural invasion (VPI), which was further defined as invasion beyond the elastic layer (PL1), invasion into the visceral pleural surface (PL2), and invasion into any component of the parietal pleura (PL3). ^3^Others: Papillary, Mucinous, Solid, Micropapillary, Enteric. ^4^Sublobar operations include segmentectomy and wedge resection.

## 4 Discussion

We acknowledge that the retrospective reclassification of tumors using the AJCC 8th edition may not fully reflect treatment decisions at the time of diagnosis. However, it provides staging consistency across time and enables meaningful survival comparisons in the evolving context of NSCLC management.

SEER data from the U.S. show a stage migration in lung cancer, with Stage I/II diagnoses increasing from 26.5% to 31.2% and Stage III/IV decreasing from 70.8% to 66.1%. Adenocarcinoma incidence also rose from 42.9% in 2006 to 59.0% in 2016 [2]. In Taiwan, similar changes in Stage I NSCLC detection and surgical management have been driven by increased LDCT use [18, 19]. LDCT enables earlier identification of small tumors that might otherwise go undetected, shifting treatment toward earlier‐stage disease, and less invasive surgery [8, 18, 20].

The introduction of LDCT screening programs has significantly increased detection of early‐stage NSCLC, especially tumors ≤ 1 cm (Stage IA1). Our data showed a rise in IA1 cases from 12.1% in 2018 to 38.2% in 2022, highlighting LDCT's impact on early diagnosis. This has expanded opportunities for curative, less aggressive surgery and influenced treatment decision‐making [3, 4, 18, 20].

Prior to LDCT, lung cancer was often diagnosed at advanced stages requiring systemic therapies. LDCT has made curative surgery more feasible by shifting diagnosis toward Stage I disease, prompting reassessment of surgical strategies.

Consequently, lung‐sparing procedures such as segmentectomy and wedge resection have become more common for early‐stage NSCLC [15]. In our cohort, sublobar resections with RLND yielded RFS comparable to lobectomy across all subgroups. No significant RFS differences were observed among lobectomy, segmentectomy, and wedge resection.

Analysis of the NCDB (2003–2011) found sublobar resection was linked to poorer survival than lobectomy in Stage IA NSCLC [21]. However, newer studies show that for tumors ≤ 2 cm—particularly in older patients with poor lung function—sublobar resection provides comparable outcomes with fewer complications [15, 22]. Lin et al. reported similar OS and RFS between segmentectomy and lobectomy, while wedge resection was associated with worse prognosis [23].

GGO‐dominant tumors seen on CT have favorable outcomes and are suitable for sublobar resection [24, 25]. In contrast, solid‐dominant tumors are more aggressive and often involve nodal spread [26]. While lobectomy remains standard for these cases, selected patients may benefit from sublobar resection [14]. A meta‐analysis found no OS difference between sublobar resection and lobectomy (HR = 1.28), though lobectomy offered better RFS, especially for tumors > 2 cm. Segmentectomy alone provided outcomes comparable to lobectomy [27].

Our data reflect this trend: lobectomy with RLND declined from 75% in 2010 to 43.6% in 2022, while segmentectomy and wedge resection increased to 22.9% and 29.8%, respectively. This aligns with trials like JCOG0802/WJOG4607L and CALGB/Alliance 140503, which support sublobar resection for small tumors [16, 17], with segmentectomy offering comparable survival and better lung preservation [15].

Preserving lung function is critical in aging patients and those with COPD or ILD [28]. Risk factors for non‐cancer death include age ≥ 70, male sex, low BMI, low FEV1, and complications [29]. Minimally invasive procedures like segmentectomy and wedge resection improve both outcomes and quality of life [15, 17, 21], and robotic segmentectomy is increasingly used over thoracoscopic approaches for Stage IA NSCLC [30].

Systematic LND remains important for part‐solid and pure‐solid tumors [26]. Haruki et al. advised caution with selective LND in solid‐predominant lower lobe tumors [31], while Maniwa et al. supported selective LND for part‐solid but recommended full dissection for pure‐solid tumors [32]. RLND is standard in Taiwan for both lobectomy and sublobar resections.

Lobectomy remains preferred for Stage IB due to recurrence risk [33], but sublobar resection is used in high‐risk patients. Individualized surgical planning is increasingly guided by patient factors and tumor biology.

Despite broader use of sublobar resection, recurrence remains a concern. Meta‐analyses link poorer RFS to older age, male sex, advanced stage, tumor size, and geography [34]. STAS has emerged as a key prognostic factor. Our study confirmed STAS as the only independent predictor of recurrence, consistent with prior findings [35, 36]. Tumor size and pleural invasion lost significance after adjustment, likely due to collinearity with STAS. Its invasive pattern may allow spread beyond resection margins, increasing recurrence risk [37, 38].

These findings support incorporating STAS into preoperative evaluation and follow‐up planning. Future work should focus on multiomic and imaging‐based tools—like radiomics and AI—to non‐invasively predict STAS and guide personalized treatment in Stage I NSCLC.

While this study offers important insights into Stage I lung cancer management, especially surgical outcomes in a tertiary hospital in Taiwan, several limitations must be noted. As a retrospective study, it is subject to inherent biases in data collection and reporting. We lacked detailed CT‐based nodule characteristics (e.g., GGO, part‐solid, and solid patterns), limiting the evaluation of how imaging features influenced surgical decisions between sublobar and lobar resection.

Additionally, patient‐level data such as smoking status, family history, and surgeon variability were unavailable, which may have influenced treatment choices and outcomes. Future studies should incorporate comprehensive clinical, radiologic, and molecular data—including nodule density, exposure history, and surgical expertise—to better assess recurrence risk.

Another limitation is the shorter follow‐up for patients treated between 2018 and 2022, which precluded accurate assessment of RFS and OS in this group. Thus, RFS analysis was limited to patients treated from 2016 to 2018, the earliest period with available STAS data. Although modest in size, this cohort provided the most complete dataset for evaluating the prognostic impact of STAS in Stage I NSCLC.

### 4.1 Further Implications

Based on our findings, the assessment of STAS should be incorporated into routine pathological evaluation and preoperative decision‐making for patients with Stage I NSCLC. Clinicians should consider using tailored surgical strategies, potentially favoring more extensive resections or closer surgical margins for STAS‐positive tumors. In addition, patients with STAS may benefit from intensified postoperative surveillance and careful consideration for adjuvant therapy to reduce the risk of recurrence. This approach emphasizes personalized patient management and aligns treatment decisions more closely with the patient's prognostic risk, ultimately enhancing clinical outcomes and optimizing resource utilization.

In conclusion, LDCT screening has markedly improved the early detection of Stage I NSCLC, enabling an increase in less invasive surgical interventions. Sublobar resections including segmentectomy and wedge resection offered survival outcomes comparable to lobectomy for patients with Stage I NSCLC and its subgroups in our real‐world cohort. The identification of STAS as a significant risk factor for recurrence underscores the critical importance of individualized risk assessment for patients with Stage I NSCLC. Further research is urgently needed to optimize adjuvant therapy for patients with Stage I NSCLC with STAS.

## Ethics Statement

The study was conducted in accordance with the Declaration of Helsinki and approved by the Institutional Review Board of Kaohsiung Medical University Hospital (KMUH) (KMUHIRB‐E(I)‐20240126).

## Conflicts of Interest

The authors declare no conflicts of interest.

## Supporting information


**Figure S1.** Flowchart for the cohort study.

## Data Availability

The data that support the findings of this study are available on request from the corresponding author. The data are not publicly available due to privacy or ethical restrictions.
