# Supplementary material for: Impact of Spread Through Air Spaces (STAS) on Recurrence and Surgical Trends in Stage I Non‐Small Cell Lung Cancer: A Real‐World Cohort Study
Source: Kaohsiung J Med Sci. 2025 Jun 17;41(10):e70061. doi: 10.1002/kjm2.70061 (PMC12520515; doi:10.1002/kjm2.70061)
Supplement: Supplementary file 1 — Figure S1. Flowchart for the cohort study. [file KJM2-41-e70061-s001.pdf]

1. Stage I NSCLC 2010 to 2017 (AJCC 7th edition) n=437
Stage I NSCLC
Stage IA :Tumor size ≤ 3 cm)
Stage IB :Tumor size > 3 cm but ≤ 5 cm)
additional features such as visceral pleural invasion or obstructive pneumonitis
2. Stage I NSCLC 2018 to 2022 (AJCC 8th edition) n=640
Stage IA1: Tumor size ≤ 1 cm
Stage IA2: Tumor size > 1 cm but ≤ 2 cm
Stage IA3: Tumor size > 2 cm but ≤ 3 cm
Stage IB: Tumor size > 3 cm but ≤ 4 cm, or any tumor size with invasion into the visceral pleura or atelectasis/obstructive pneumonitis.

Data were collected from the hospital records of NSCLC patients from 2010 to 2022.Data from the Taiwan National Cancer Registry through 2022 were also obtained to ensure accurate staging and survival outcomes

Stage I subgroup distribution in 2010 to 2017 (AJCC 7th edition) and 2018 to 2022 (AJCC 8th edition)
Trend of surgery as the first-line treatment for NSCLC Stages I to IV
Trend in surgical interventions for NSCLC Stage 1A and IB (2010-2017)
Trend in surgical interventions for NSCLC Stage IA1, IA2, IA3, and IB (2018-2022)
N = 1077

Patients were included if they had histologically confirmed Stage I NSCLC, underwent surgical resection, and had complete follow-up data.
The exclusion criteria were missing pathology data, neoadjuvant therapy, non-curative surgery, or loss to follow-up before recurrence was diagnosed.

Cox regression model for recurrence free survival (RFS) in stage I NSCLC patients diagnosed between January 2016 and December 2018, median follow-up time 63.7 months. STAS data were only recorded in the cancer registry beginning in 2016. N=207
